# Supplementary material for: From sequence to enzyme mechanism using multi-label machine learning
Source: BMC Bioinformatics. 2014 May 19;15:150. doi: 10.1186/1471-2105-15-150 (PMC4229970; doi:10.1186/1471-2105-15-150)
Supplement: Additional file 2 — Java code of ml2db. Additional file ml2db_code.tar.gz contains the Java source code to run the multi-label machine learning experiments and save the results to database. The code’s Javadoc is included. [file 1471-2105-15-150-S2.zip › additional file 2/ml2db/utils/doc/uk/ac/standrews/utils/main/database/class-use/Manager.html]

Uses of Class uk.ac.standrews.utils.main.database.Manager


JavaScript is disabled on your browser.


- Overview
- Package
- Class
- Use
- Tree
- Deprecated
- Index
- Help

- Prev
- Next

- Frames
- No Frames

- All Classes

## Uses of Class uk.ac.standrews.utils.main.database.Manager

- Packages that use Manager

  | Package | Description |
  | --- | --- |
  |  |  |
  | --- | --- |
  | uk.ac.standrews.utils.main.database |  |
- - ### Uses of Manager in uk.ac.standrews.utils.main.database

    Subclasses of Manager in uk.ac.standrews.utils.main.database

    | Modifier and Type | Class and Description |
    | --- | --- |
    |  |  |
    | --- | --- |
    | `class` | `DbManager` A database manager provides object to create a database, write and read from it. |
    | `class` | `TableCreator` Class to create a table object in the database. |
    | `class` | `TableManager` Class to create/read/write to a database table. |
    | `class` | `TableReader` Class |
    | `class` | `TableRow` Contains the data for a generic row (a list of [table column, value] entries) to be written to a database table. |
    | `class` | `TableWriter` A TableWriter object contains all the information to create, fill and delete a database table. |

    Methods in uk.ac.standrews.utils.main.database that return Manager

    | Modifier and Type | Method and Description |
    | --- | --- |
    |  |  |
    | --- | --- |
    | `Manager` | Managed.`getManager()` |

    Methods in uk.ac.standrews.utils.main.database with parameters of type Manager

    | Modifier and Type | Method and Description |
    | --- | --- |
    |  |  |
    | --- | --- |
    | `void` | Managed.`setManager(Manager manager)` |

    Constructors in uk.ac.standrews.utils.main.database with parameters of type Manager

    Constructor and Description

    `Managed(Manager manager)`

- Overview
- Package
- Class
- Use
- Tree
- Deprecated
- Index
- Help

- Prev
- Next

- Frames
- No Frames

- All Classes
